# The Prevalence of Attention Deficit/Hyperactivity Disorder among Chinese Children and Adolescents

**DOI:** 10.1038/s41598-018-29488-2

**Published:** 2018-08-16

**Authors:** Anni Liu, Yunwen Xu, Qiong Yan, Lian Tong

**Affiliations:** 1 School of Public Health, Fudan University/Key Laboratory Public Health Safety, Chinese Ministry of Education, Shanghai, China; 2 Department of Epidemiology, Johns Hopkins University Bloomberg School of Public Health, Baltimore, Maryland United States

## Abstract

Updating the worldwide prevalence estimates of attention-deficit hyperactivity disorder (ADHD) has significant applications for the further study of ADHD. However, previous reviews included few samples of Chinese children and adolescents. To conduct a systematic review of ADHD prevalence in Mainland China, Hong Kong, and Taiwan to determine the possible causes of the varied estimates in Chinese samples and to offer a reference for computing the worldwide pooled prevalence. We searched for PubMed, Embase, PsycINFO, Web of Science, China National Knowledge Infrastructure, VIP, WANFANG DATA, and China Science Periodical Database databases with time and language restrictions. A total of 67 studies covering 642,266 Chinese children and adolescents were included. The prevalence estimates of ADHD in Mainland China, Hong Kong, and Taiwan were 6.5%, 6.4%, and 4.2%, respectively, with a pooled estimate of 6.3%. Multivariate meta-regression analyses indicated that the year of data collection, age, and family socioeconomic status of the participants were significantly associated with the prevalence estimates. Our findings suggest that geographic location plays a limited role in the large variability of ADHD prevalence estimates. Instead, the variability may be explained primarily by the years of data collection, and children’s socioeconomic backgrounds, and methodological characteristics of studies.

## Introduction

Attention-deficit/hyperactivity disorder (ADHD) is one of the most common childhood psychiatric disorders, with symptoms including inattention, impulsivity, and hyperactivity^1–3^. As a major public health problem^4^, ADHD has been associated with a wide variety of adverse health outcomes for affected individuals^5,6^ and severe financial burdens for families and societies^7^. Concerns have been raised regarding the true prevalence of ADHD among children, the knowledge of which is critical for further service planning, resource allocation, training, and research priorities^8^. In the last few decades, a host of investigators have made substantial efforts to determine the prevalence of ADHD. Several reviews reported a broad range of prevalence rates, from as low as nearly 1% to as high as nearly 20% among children and adolescents throughout the world^9–14^. A comprehensive review including 102 studies worldwide reported a pooled prevalence estimate of 5.3% in children^11^. Another review covering 86 studies found that the prevalence estimates only employing the Diagnostic and Statistical Manual of Mental Disorders, Fourth Edition (DSM-IV) as the diagnostic criteria varied from 5.9% to 7.1% in children and adolescents^14^. However, previous systematic reviews seldom selected a sufficient proportion of studies conducted among Asian children and adolescents, and were especially lacking of Chinese samples, despite the fact that China has the largest number of children and adolescents in the world.

To our knowledge, the first investigation of Minor Brain Dysfunction (an alternative name of ADHD) prevalence among children was conducted in Mainland China in 1981^15^. With the introduction of the Diagnostic and Statistical Manual of Mental Disorders, Third Edition (DSM-III) into China in the 1980s, multifold epidemiological surveys have been carried out on children and adolescents in Mainland China, Hong Kong, and Taiwan, and yielded the prevalence estimates ranging from as low as 0.7% to as high as 14.1%^16,17^. Our previous systematic review published in Chinese included 33 studies conducted in Mainland China from 1983 to 2011 and reported a pooled ADHD prevalence of 5.7% in Chinese children and adolescents^18^.

Geographical, demographic and cultural factors have been suggested as important variables that contribute to the heterogeneity of ADHD prevalence across studies^12,19^. Given that the scope of this systematic review falls on the Chinese children and adolescents in Mainland China, Hong Kong and Taiwan, it is noteworthy to mention the features of social-cultural contexts in those three regions. For instance, compared with Hong Kong and Taiwan, the children in Mainland China have experienced more intensely unbalanced development, due to historical reasons and social transformations^20^. Furthermore the significant gaps in the economic situations among different Urban and rural areas within the Mainland China may fuel the diverse epidemiological aspects of ADHD. More importantly, the long-term one-child policy has notably affected the children’s living surroundings in Mainland China, and thus may increase the chances of suffering the psychological and behavioral problems for the only children due to the lack of playmates, compared to the children with siblings^21,22^. It is also worth mentioning that the highly competitive educational system with the increasing academic pressures in China may expose Chinese children to chronic stress, thus increasing susceptibility to mental health problems, including ADHD^23,24^. Methodological characteristics, such as screening and diagnostic methods, may be associated with the heterogeneity in prevalence results as well^5,12^.

It is clear that an estimated ADHD prevalence from one location fails to represent the overall prevalence among Chinese children, while a systematic understanding of the ADHD prevalence estimates in Chinese children and adolescents may provide a better insight into the overall and subgroup distribution and etiology of ADHD under different social and cultural backgrounds. Furthermore, a meta-analysis that computes the prevalence estimates of ADHD in the three regions will offer the supportive data for the accurate prediction of the worldwide pooled prevalence. Therefore, the purposes of this study are: (1) to estimate the overall and subgroup prevalence estimates of ADHD among children and adolescents in Mainland China, Hong Kong, and Taiwan from 1980 to 2016; (2) to analyze the trends of ADHD prevalence in the three locations in a period spanning the past 3 decades to aid in predicting future trends; and (3) to explore the possible causes of the varied prevalence estimates.

## Results

### Systematic review

**Figure 1:**
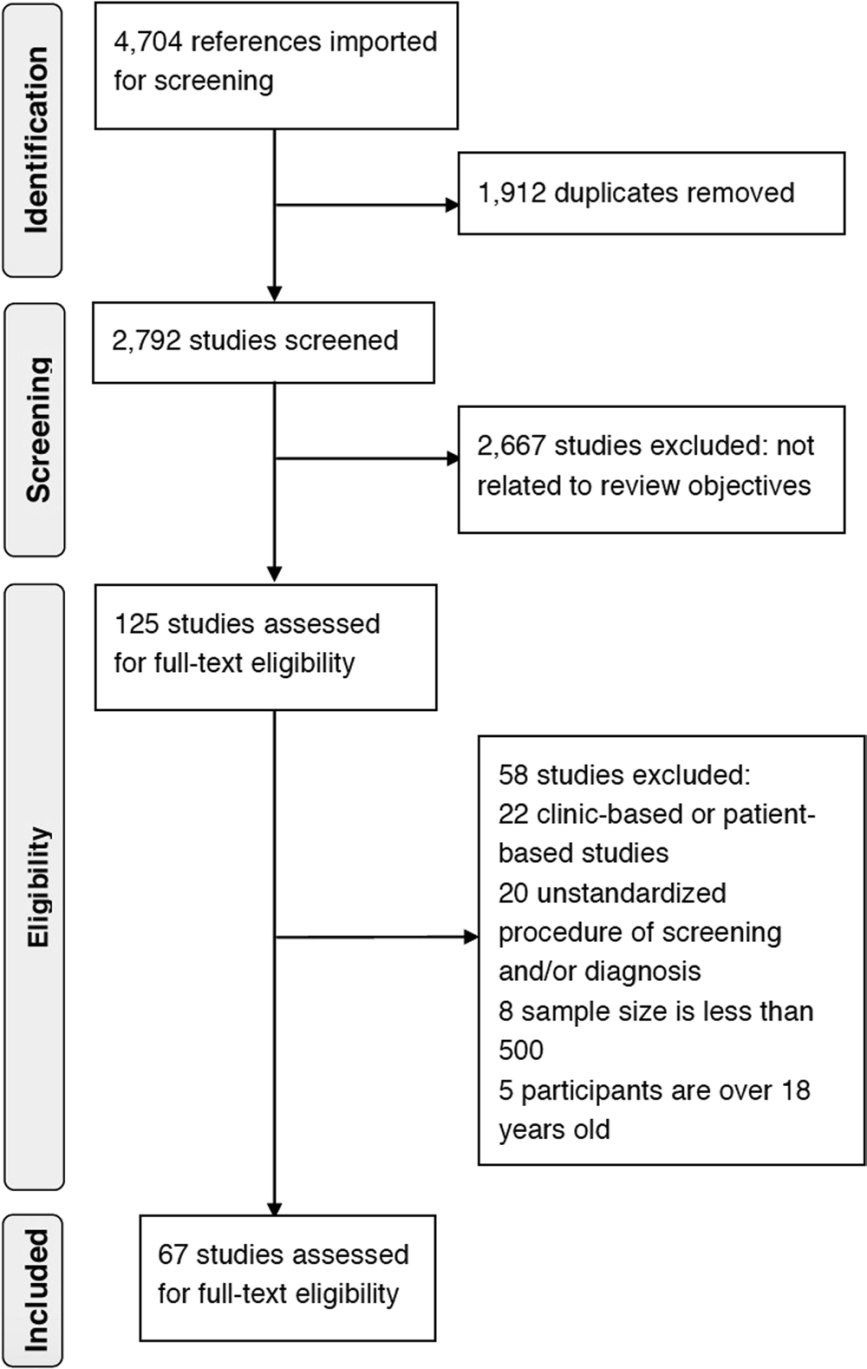
Study selection flowchart.

We screened 4704 abstracts, reviewed 125 full-text articles, and selected 67 studies for the final systematic review. Of these, 13 were published in English and 54 were published in Chinese. Figure 1 presents the flowchart of study selection. Table 1 displays the characteristics of the articles included in this systematic review. A total of 70 ADHD prevalence rates were reported in the 67 studies. Specifically, 60 prevalence rates were from Mainland China, 2 from Hong Kong, and 8 from Taiwan (Table 1). A total of 642266 children and adolescents were included in our systematic review, 227943 of them being from Mainland China, 3610 from Hong Kong, and 410713 from Taiwan. The first epidemiological investigation of Chinese children with ADHD was conducted in Mainland China in 1981^15^. Only 6 studies (9.0%) were conducted in the first 10 years (1980–1990), and the number of studies remarkably increased from 1991–2000 (10 studies) to 2001–2010 (33 studies). In recent years (2011–2016), 18 studies were carried out. In terms of regions, 49 prevalence rates were determined from samples collected in urban areas, 20 were based on mixed samples from both Urban,& rural areas, and only 1 focused solely on children in rural areas. The time frame of data collection varied across studies, indicating different study designs. The majority of the prevalence rates were from cross-sectional studies, 59 of which collected the data within 1 year. Five cohort studies in Taiwan implemented the data collection for over 2 years. Only 7 studies chose preschoolers as the study population, while over 60% of studies consisted of school-aged children and adolescents. Among the 67 studies, the Conners’ Parent Rating Scale and Conners’ Teacher Rating Scale were the most commonly used screening methods. Over half of the studies employed the diagnostic criteria of the DSM-III/III-R/IV.

**Table 1:** Description of studies included in the systematic review.

Author, y	City/Location	Time Frame	Region	Sample’s Age	Proportion of Boys	Sample Size	Source of Information	Screening and/or Diagnosis	Screening Criteria	Diagnostic Criteria	Original Prevalence	Risk of Bias Score
Wang RC *et al*.^41^ 1983	Baoding	≤1 year	Urban	School age	0.51	1,588	T	Screening, & diagnosis	Others^a^	DSM-III	0.03	5
Tang WB *et al*.^42^ 1987	Tianjin	≤1 year	Urban	School age	—	9,971	T and P	Screening, & diagnosis	Others^a^	DSM-III	0.04	6
Zhang ML *et al*.^17^ 1991	Baotou	≤1 year	Urban, & rural	School age	0.53	14,739	T and P	Screening, & diagnosis	Conners, & Others^a^	DSM-III, & Others^a^	0.14	7
Wang LM *et al*.^43^ 1993	Harbing	≤1 year	Urban	School age	0.51	1,377	T and P	Screening, & diagnosis	Conners, & DSM-III	DSM-III	0.07	7
Tang JP *et al*.^44^ 1993	Changsha	≤1 year	Urban	Preschool, & school age	—	1,173	T and P	Screening, & diagnosis	Others^a^	CCMD-II	0.04	5
Lin YL *et al*.^45^ 1996	Putian	≤1 year	Urban, & rural	School age	0.51	12,638	T and P	Screening, & diagnosis	Conners, & Others^a^	CCMD-II-R	0.03	7
Zhang JP *et al*.^46^ 1999	Hefei	≤1 year	Urban, & rural	School age	0.49	1,021	P	Screening	Others^a^	—	0.11	6
Jiang H *et al*.^47^ 2000	Shanghai	≤1 year	Urban, & rural	School age	0.49	1,310	T	Screening	Conners, & Others^a^	—	0.04	5
Jiang L *et al*.^48^ 2002	Zhenjiang	≤1 year	Urban	School age	0.49	3,698	P	Screening	DSM-IV	—	0.07	8
Wang XL *et al*.^49^ 2002	Xiamen	1–2 years	Urban	School age	0.50	3,989	T and P	Screening, & diagnosis	Conners	DSM-IV, & Others^a^	0.06	7
Sun XY *et al*.^50^ 2003	Zibo	≤1 year	Urban, & rural	Preschool, & school age	0.53	3,987	T and P	Screening, & diagnosis	Conners	DSM-III	0.04	7
Chen SZ *et al*.^51^ 2004	Guilin	≤1 year	Urban	Preschool, & school age	0.49	9,162	T and P	Screening, & diagnosis	Conners, & DSM-IV	DSM-IV	0.04	5
Ying WG *et al*.^52^ 2004	Heze	≤1 year	Urban	School age	0.52	912	T and P	Diagnosis	—	CCMD-III	0.08	5
Kulibahan *et al*.^53^ 2005	Kuitun	≤1 year	Urban	School age	0.57	1,244	T and P	Screening, & diagnosis	Conners	DSM-IV	0.12	7
Huangfu ZM *et al*.^54^ 2006	Foshan	≤1 year	Urban	School age	0.50	2,982	P	Screening	DSM-IV	—	0.02	7
Liu L *et al*.^55^ 2006	Ningxia	≤1 year	Urban	School age	0.51	2,664	T	Screening	Conners	—	0.13	7
Zhang W *et al*.^56^ 2007	Six cities in Mainland China	≤1 year	Urban, & rural	School age	0.49	1,051	P	Screening	DSM-IV	—	0.05	6
Yang BF *et al*.^57^ 2007	Jining	≤1 year	Urban, & rural	School age	0.51	1,158	P	Screening	Conners	—	0.07	7
Ba JF *et al*.^58^ 2008	Huaibei	≤1 year	Urban	School age	0.55	2,141	T	Screening	Conners	—	0.08	7
Sun D *et al*.^59^ 2008	Mudanjiang	≤1 year	Urban	School age	0.56	6,994	P	Screening, & diagnosis	Conners	CCMD-III	0.09	7
Sun DF *et al*.^60^ 2008	Shandong	≤1 year	Urban	Preschool, & school age	0.51	8,235	P	Screening, & diagnosis	DSM-IV	Others^a^	0.06	7
Jiang H *et al*.^61^ 2008	Weihai	≤1 year	Urban	School age	0.53	4,268	P	Screening	DSM-IV	—	0.06	7
Chang XL *et al*.^62^ 2009	Zhenjiang	≤1 year	Urban, & rural	Preschool age	0.51	724	P	Screening	Conners	—	0.03	7
Han LT *et al*.^63^ 2010	Liaoyang	≤1 year	Urban	School age	0.50	5,000	T and P	Screening, & diagnosis	Conners, DSM-IV, & Others^a^	DSM-IV	0.12	6
Zhang BC *et al*.^16^ 2011	Guiyang	≤1 year	urban	School age	0.47	3,016	P	Screening, & diagnosis	Conners	DSM-IV	0.01	7
Wang HM *et al*.^64^ 1997	Taiyuan	≤1 year	Urban	School age	0.50	2,114	T and P	Screening	DSM-III-R	—	0.04	7
Zhao PF *et al*.^65^ 2005	Shaodong	≤1 year	Urban, & rural	School age	0.51	1,069	—	Screening	Conners	—	0.09	7
Xu M *et al*.^66^ 2005	Fuan	≤1 year	Urban	Preschool, & school age	0.51	3,738	P	Screening	DSM-III	—	0.04	5
Liang D *et al*.^67^ 2006	Changchun	≤1 year	Urban	School age	0.49	7,117	T	Screening	Conners	—	0.02	7
Liang D *et al*.^67^ 2006	Changchun	≤1 year	Urban	School age	0.49	7,117	P	Screening	Conners	—	0.01	7
Tang SW *et al*.^68^ 2008	Urumuqi	≤1 year	Urban	Preschool age	0.49	1,967	T	Screening	Conners	—	0.08	7
Shi ST *et al*.^69^ 2002	Yunnan	1–2 years	Urban, & rural	School age	0.50	5,650	T and P	Screening	Conners. & DSM-IV	—	0.07	5
Guo M *et al*.^70^ 2008	Nanchang	≤1 year	Urban	School age	0.54	633	T and P	Screening,& diagnosis	Conners	CCMD-III	0.06	5
Guan BQ *et al*.^71^ 2005	Six cities in Mainland China	≤1 year	Urban, & rural	School age	0.54	9,495	T and P	Screening, & diagnosis	DSM-IV	DSM-IV, & Others^a^	0.06	8
Guo HL *et al*.^72^ 2011	Binzhou	≤1 year	Urban	School age	0.43	4,275	P	Screening	DSM-IV, & Others^a^	—	0.06	6
Wang SY *et al*.^73^ 2014	Lanzhou	≤1 year	Urban	School age	0.51	3,604	T, & P	Screening, & diagnosis	Conners	DSM-IV	0.11	7
Xu GQ *et al*.^74^ 2012	Cixi	1–2 years	Urban	Preschool, & school age	—	1,245	—	Screening	DSM-IV	—	0.08	5
Liu F *et al*.^75^ 2012	Liuzhou	1–2 years	Urban, & rural	School age	0.50	1,021	P	Screening, & diagnosis	DSM-IV	DSM-IV	0.04	6
Yu L *et al*.^76^ 2013	Huizhou	≤1 year	Urban	School age	0.47	6,856	P	Screening, & diagnosis	DSM-IV	DSM-IV	0.07	5
Wang LZ *et al*.^77^ 2010	Wuxi	≤1 year	Urban	Preschool age	0.56	604	P	Screening, & diagnosis	Conners	DSM-IV	0.05	6
Zhang HY *et al*.^78^ 2010	Lanzhou	≤1 year	Urban	Preschool, & school age	0.55	1,001	P	Screening, & diagnosis	Conners	DSM-IV	0.09	5
Li Y *et al*.^79^ 2015	Tianjin	≤1 year	Urban	Preschool, & school age	0.59	2,046	P	Screening, & diagnosis	DSM-IV	DSM-IV, & Conners	0.14	7
Jiang HJ *et al*.^80^ 2013	Dongyang	≤1 year	Urban	Preschool, & school age	0.53	3,882	P	Screening	Conners	—	0.05	6
Shi LJ *et al*.^81^ 2012	Leshan	≤1 year	Urban	Preschool, & school age	0.52	1,400	P	Screening	DSM-IV	—	0.04	7
Zhang CJ *et al*.^82^ 2014	Guiyang	≤1 year	Urban	Preschool age	0.54	4,489	T and P	Screening, & diagnosis	DSM-IV	Others^a^	0.01	7
Wang AP *et al*.^83^ 2011	Yiwu	≤1 year	Urban	Preschool, & school age	—	1,376	—	Screening	DSM-IV	—	0.09	7
Meng LP *et al*.^84^ 1999	Jiaozuo	≤1 year	Urban	Preschool, & school age	0.54	904	P	Screening	DSM-III-R	—	0.10	7
Shen P,^85^ 2012	Wuxi	≤1 year	Urban	School age	0.54	2,397	S	Screening	CCMD-III	—	0.10	8
Wang C *et al*.^86^ 1982	Hengyang	≤1 year	Urban	School age	0.50	3,804	T and P	Diagnosis	—	DSM-III	0.07	5
Li XL *et al*.^87^ 2012	Wulumuqi	≤1 year	Urban	School age	0.53	2,066	T and P	Screening, & diagnosis	Conners	DSM-IV, & Conners	0.05	7
Luo Z *et al*.^88^ 2013	Taizhou	≤1 year	Rural	Preschool age	—	1,699	P	Screening	Conners	—	0.12	6
He M *et al*.^89^ 2012	Guangzhou	≤1 year	Urban	Preschool age	0.55	1,326	T and P	Screening, & diagnosis	Conners	DSM-IV	0.05	6
Jin WL *et al*.^90^ 2009	Shanghai	≤1 year	Urban	Preschool, & school age	0.50	5,648	T and P	Screening, & diagnosis	DSM-IV	DSM-IV	0.05	6
Leung PW *et al*.^91^ 2008	Hong Kong	≤1 year	Urban	School age	0.48	541	P and S	Screening	DISC-IV, & Others^a^	—	0.04	10
GauSS *et al*.^92^ 1995	Taiwan	≤1 year	Urban, & rural	School age	0.50	1,070	T	Screening, & diagnosis	Others^a^	DSM-IV	0.08	7
GauSS *et al*.^92^ 1996	Taiwan	≤1 year	Urban, & rural	School age	0.50	1,051	T	Screening, & diagnosis	Others^a^	DSM-IV	0.06	7
GauSS *et al*.^92^ 1997	Taiwan	≤1 year	Urban, & rural	School age	0.50	1,035	T	Screening, & diagnosis	Others^a^	DSM-IV	0.03	7
Leung PW *et al*.^93^ 1996	Hong Kong	≤1 year	Urban	School age	1.00	3,069	T and P	Screening, & diagnosis	Others^a^	DSM-III-R	0.09	7
Lu L *et al*.^94^ 2003	Wuhan	≤1 year	Urban	Preschool, & school age	0.49	2,128	T and P	Screening, & diagnosis	Conners, & DSM-IV	Others^a^	0.14	7
Lam LT *et al*.^95^ 2005	Nanning	≤1 year	Urban	School age	0.47	1,429	S	Diagnosis	—	DSM-IV, & Conners	0.08	8
Chien IC *et al*.^96^ 1996	Taiwan	>2 years	Urban, & rural	Preschool, & school age	—	372,642	—	Diagnosis	—	ICD-9-CM	0.02	8
Xiaoli Y *et al*.^24^ 2008	Six cities in Mainland China	≤1 year	Urban, & rural	School age	0.53	8,848	T, P and S	Screening, & diagnosis	Others^a^	Others^a^	0.01	9
Shen YC *et al*.^97^ 1985	Beijing	≤1 year	Urban & rural	School age	0.51	2,770	T	Screening, & diagnosis	Others^a^	DSM-III	0.06	8
Tseng WL *et al*.^98^ 2008	Taiwan	≤1 year	Urban	School age	0.52	739	P	Screening	DSM-IV	—	0.08	8
Provincial psychiatric hospital *et al*.^15^ 1980	Guiyang	≤1 year	Urban	School age	0.51	4,142	—	Diagnosis	—	Others^a^	0.06	7
Qu Y *et al*.^99^ 2013	Four cities in Mainland China	1–2 years	Urban, & rural	School age	0.50	19,711	P and S	Screening, & diagnosis	Others^a^	DSM-IV, & Others^a^	0.05	9
Ko WR *et al*.^100^ 2005	Taiwan	>2 years	Urban	Preschool, & school age	0.66	13,172	—	Diagnosis	—	ICD-9-CM	0.04	8
Xie YR *et al*.^101^ 1981	Guilin	≤1 year	Urban	School age	0.51	2,447	T and P	Screening, & diagnosis	Others^a^	DSM-III	0.07	7
Chen MH *et al*.^102^ 2000	Taiwan	>2 years	Urban	Preschool age	0.60	9,176	—	Diagnosis	—	ICD-9-CM	0.05	8
Chen MD *et al*.^103^ 1996	Taiwan	>2 years	Urban	Preschool, & school age	0.36	11,828	—	Diagnosis	—	ICD-9-CM	0.02	8

Conners,Conners’ Parent Rating Scale and/or Conners’ Teacher Rating Scale; DSM-III, Diagnostic and Statistical Manual of Mental Disorders, Third Edition; DSM-III-R, Diagnostic and Statistical Manual of Mental Disorders, Revised Third Edition; DSM-IV, Diagnostic and Statistical Manual of Mental Disorders, Fourth Edition; CCMD-II, Chinese Classification and Diagnosis of Mental Diseases, Second Edition; CCMD-II-R, Chinese Classification and Diagnosis of Mental Diseases, Revised Second Edition; CCMD-III, Chinese Classification and Diagnosis of Mental Diseases, Third Edition; ICD-9-CM, The International Classification of Diseases, Ninth Revision, Clinical Modification; DISC-IV, Diagnostic Interview Schedule for Children-Version 4; —, no available data or not applicable; T, teacher; P, parent; S, student.

^a^Others appearing in both screening criteria and diagnostic criteria columns means the authors employed other types of screening criteria or diagnostic criteria, such as clinical checks, interviews, and standard questionnaires (e.g., Eysenck Personality Questionnaire, Achenbach’s child behavioral checklist, Rutter’s Teacher (B2) Questionnaire and Parent (A3) Questionnaire, Standardized Chinese Version of the Child Behavior Checklist, etc.) in their prevalence studies.

Although all estimates from 67 studies were at moderate or low risk of bias, only 1 estimate met all 10 criteria, and 65% were at low risk of bias. The majority of estimates rated poorly for the representativeness of the national population (93%), and the strict measurement of the reliability and validity of the study instrument (85%). Besides, most estimates did not collect ADHD diagnostic information directly from children or adolescents (93%). Summary statistics for risk of bias for estimates are provided in Table 1.

### Prevalence of ADHD

**Figure 2:**
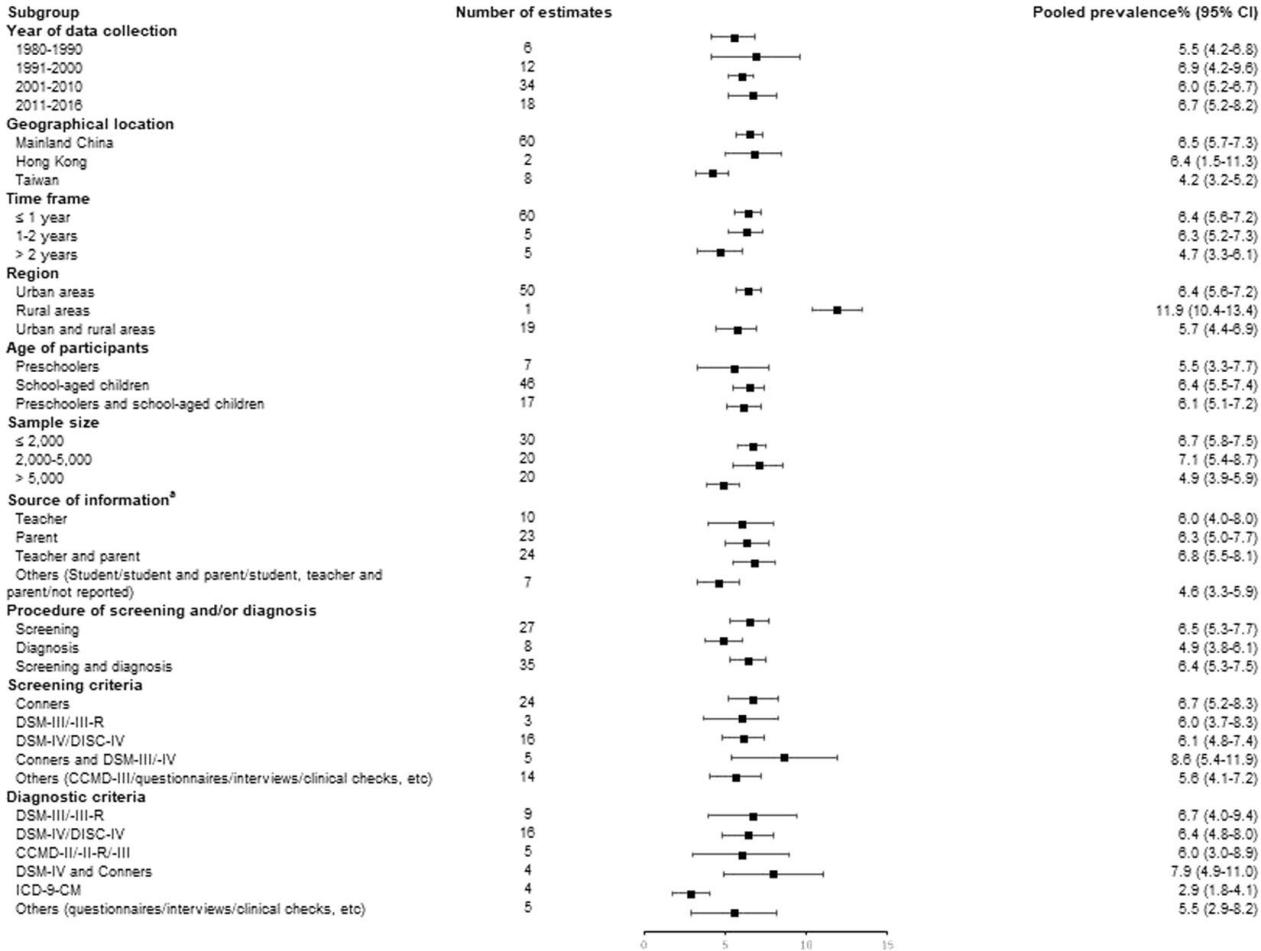
Forest plot of subgroup prevalence estimates among children and adolescents in three regions. ^a^Data was not available in some studies.

The overall and subgroup prevalence estimates of ADHD are shown in Table 2. The pooled prevalence of ADHD was 6.3% (95% confidence interval [CI], 5.7–6.9). The estimated rates in Mainland China, Hong Kong, and Taiwan were 6.5% (95% CI, 5.7–7.3), 6.4% (95% CI, 1.5–11.3), and 4.2% (95% CI, 3.2–5.2), respectively. ADHD was more common in boys (8.9%, 95% CI, 7.6–10.2) than in girls (4.0%, 95% CI, 3.4–4.7). The pooled prevalence rates were 5.5% (95% CI, 4.2–6.8) between 1980 and 1990, 6.9% (95% CI 4.2–9.6) between 1991 and 2000, 6.0% (95% CI, 5.2–6.7) between 2001 and 2010, and 6.7% (95% CI, 5.2–8.2) between 2011 and 2016. The pooled prevalence rate was 5.5% (95% CI, 3.3–7.7) for preschoolers, and 6.5% (95% CI, 5.5–7.4) for school-aged children and adolescents, while the overall prevalence of combining two age groups was 6.1% (95% CI, 5.1–7.2). The forest plot of the subgroup estimates is presented in Fig. 2.

**Table 2:** General characteristics of included prevalence rates.

Study characteristics	Number of estimates	Prevalence estimates (%)	95% CI of estimates
**Year of data collection**			
1980–1990	6	5.5	4.2–6.8
1991–2000	12	6.9	4.2–9.6
2001–2010	34	6.0	5.–6.7
2011–2016	18	6.7	5.22–8.2
**Geographical location**			
Mainland China	60	6.5	5.7–7.3
Hong Kong	2	6.4	1.5–11.3
Taiwan	8	4.2	3.2–5.2
**Time frame**			
≤1 year	60	6.4	5.6–7.2
1–2 years	5	6.3	5.2–7.3
>2 years	5	4.7	3.3–6.1
**Region**			
Urban areas	50	6.4	5.6–7.2
Rural areas	1	11.9	10.4–13.4
Urban, & rural areas	19	5.7	4.4–6.9
**Age of participants**			
Preschoolers	7	5.5	3.3–7.7
School–aged children and adolescents	46	6.4	5.5–7.4
Preschoolers, & school–aged children and adolescents	17	6.1	5.1–7.2
**Sample size**			
≤2,000	30	6.7	5.8–7.5
2,000–5,000	20	7.1	5.4–8.7
>5,000	20	4.9	3.9–5.9
**Source of information** ^**a**^			
Teacher	10	6.0	4.0–8.0
Parent	23	6.3	5.0–7.7
Teacher, & parent	24	6.8	5.5–8.1
Others (Student/student, & parent/student, teacher, & parent/not reported)	7	4.6	3.3–5.9
**Procedure of screening and/or diagnosis**			
Screening	27	6.5	5.3–7.7
Diagnosis	8	4.9	3.8–6.1
Screening, & diagnosis	35	6.4	5.3–7.5
**Screening criteria**			
Conners	24	6.7	5.2–8.3
DSM–III/–III–R	3	6.0	3.7–8.3
DSM–IV/DISC–IV	16	6.1	4.8–7.4
Conners, & DSM–III/–IV	5	8.6	5.4–11.9
Others (CCMD–III/questionnaires/interviews/clinical checks, etc.)	14	5.6	4.1–7.2
**Diagnostic criteria** ^**b**^			
DSM–III/–III–R	9	6.7	4.0–9.4
DSM–IV/DISC-IV	16	6.4	4.8–8.0
CCMD–II/–II–R/–III	5	6.0	3.0–8.9
DSM–IV, & Conners	4	7.9	4.9–11.0
ICD–9–CM	4	2.9	1.8–4.1
Others (questionnaires/interviews/clinical checks, etc.)	5	5.5	2.9–8.2
**Total**	70	6.3	5.7–6.9

CI: Confidence Interval; Conners, Conners’ Parent Rating Scale and/or Conners’ Teacher Rating Scale; DSM-III, Diagnostic and Statistical Manual of Mental Disorders, Third Edition; DSM-III-R, Diagnostic and Statistical Manual of Mental Disorders, Revised Third Edition; DSM-IV, Diagnostic and Statistical Manual of Mental Disorders, Fourth Edition; CCMD-II, Chinese Classification and Diagnosis of Mental Diseases, Second Edition; CCMD-II-R, Chinese Classification and Diagnosis of Mental Diseases, Revised Second Edition; CCMD-III, Chinese Classification and Diagnosis of Mental Diseases, Third Edition; ICD-9-CM, The International Classification of Diseases, Ninth Revision, Clinical Modification; DISC-IV, Diagnostic Interview Schedule for Children-Version 4.

^a^Some articles didn’t report the source of information.

^b^Only articles that reported the diagnostic criteria were counted.

### Sources of variability in prevalence estimates

Substantial heterogeneity across studies was detected (*I*^2^ = 99%; *Q* = 9121.98, *df* = 69, *P* < 0.001), thus, the meta-regression analyses were used to explore the potential causes. In univariate meta-regression analyses (Table 3), there was a significant increase in prevalence estimates in all three periods of 1991–2000 (*β* = 0.39, *P* < 0.001), 2001–2010 (*β* = 0.36, *P* < 0.001), and 2011–2016 (*β* = 0.38, *P* < 0.001) compared with the period of 1980–1990. The studies with combined samples from both Urban, & rural areas yielded significantly higher ADHD prevalence estimates than those with urban samples (*β* = 0.35, *P* < 0.001). Both school-aged children and adolescents (*β* = 0.37, *P* < 0.001) and preschoolers combined with school-aged children and adolescents (*β* = 0.37, *P* < 0.001) had significantly higher prevalence estimates than preschoolers. The larger sample sizes of 2000–5000 (*β* = 0.39, *P* < 0.001) or over 5,000 (*β* = 0.32, *P* < 0.001) generated significantly higher prevalence estimates than the sample sizes of less than 2000. There was a significant increase in prevalence estimates when the informants were parents (*β* = 0.37, *P* < 0.001) as well as both teachers and parents (*β* = 0.39, *P* < 0.001) compared with only teachers. The studies that underwent diagnostic procedure (*β* = 0.33, *P* < 0.001) or both screening and diagnostic procedures (*β* = 0.37, *P* < 0.001) displayed significantly higher prevalence estimates than those only with screening procedure. The studies employing Conners-based screening criteria yielded significantly lower estimates than those with other screening criteria, e.g., DSM-III/-III-R (*β* = 0.37, *P* = 0.019), DSM-IV/DISC-IV (*β* = 0.37, *P* < 0.001), Conners combined with DSM criteria (*β* = 0.44, *P* < 0.001). Compared to the studies conducted with the diagnostic criteria of DSM-III/-III-R, the studies using DSM-IV/DISC-IV (*β* = 0.37, *P* < 0.001), CCMD-II/II-R/III (*β* = 0.36, *P* = 0.001), DSM-IV combined with Conners (*β* = 0.42, *P* < 0.001) or ICD-9-CM (*β* = 0.25, *P* = 0.02) as the diagnostic criteria had significantly higher prevalence estimates.

**Table 3:** The associations between study characteristics and the ADHD prevalence estimates.

Characteristics of studies	β	95% CI	F	I²-res (%)
Years of data collection (1980–1990 as index)			130.58*	99.15
1991–2000	0.39*	0.30–0.47		
2001–2010	0.36*	0.31–0.41		
2011–2016	0.38*	0.31–0.45		
Geographical location (Mainland China as index)			3.92*	99.80
Hong Kong	0.38	−0.14–0.90		
Taiwan	0.31*	0.05–0.57		
Time frame (≤1 year as index)			4.56*	99.78
1–2 years	0.38*	0.05–0.71		
>2 years	0.31	−0.01–0.64		
Region (urban areas as index)			11.46*	99.75
Rural areas	0.53	−0.14–1.20		
Urban, & rural areas	0.35*	0.20–0.50		
Age of participants (preschoolers as index)			189.18*	98.92
School–aged children and adolescents	0.37*	0.33–0.42		
Preschoolers, & school–aged children and adolescents	0.37*	0.29–0.44		
Sample size(≤2,000 as index)			33.34*	99.42
2,000–5,000	0.39*	0.27–0.51		
>5,000	0.32*	0.20–0.44		
Source of information (teacher as index)			75.62*	98.98
Parent	0.37*	0.30–0.45		
Teacher, & parent	0.39*	0.31–0.46		
Others (Student/student, & parent/student, teacher, & parent/not reported)	0.31*	0.17–0.45		
Procedure of screening and/or diagnosis (screening as index)			41.85*	99.43
Diagnosis	0.33*	0.15–0.51		
Screening, & diagnosis	0.37*	0.28–0.46		
Screening criteria (Conners as index)			18.78*	99.61
DSM–III/–III–R	0.37*	0.06–0.68		
DSM–IV/DISC-IV	0.37*	0.24–0.50		
Conners, & DSM–III/–IV	0.44*	0.20–0.68		
Others (CCMD–III/questionnaires/interviews/clinical checks, etc.)	0.35*	0.21–0.49		
Diagnostic criteria (DSM–III/–III–R as index)			18.89*	99.62
DSM-IV/DISC-IV	0.37*	0.27–0.48		
CCMD–II/–II-R/-III	0.36*	0.17–0.56		
DSM–IV, & Conners	0.42*	0.20–0.64		
ICD–9–CM	0.25*	0.03–0.47		
Others (questionnaires/interviews/clinical checks, etc.)	0.33*	0.13–0.52		

CI, Confidence Interval; Conners, Conners’ Parent Rating Scale and/or Conners’ Teacher Rating Scale; DSM-III, Diagnostic and Statistical Manual of Mental Disorders, Third Edition; DSM-III-R, Diagnostic and Statistical Manual of Mental Disorders, Revised Third Edition; DSM-IV, Diagnostic and Statistical Manual of Mental Disorders, Fourth Edition; CCMD-II, Chinese Classification and Diagnosis of Mental Diseases, Second Edition; CCMD-II-R, Chinese Classification and Diagnosis of Mental Diseases, Revised Second Edition; CCMD-III, Chinese Classification and Diagnosis of Mental Diseases, Third Edition; ICD-9-CM, The International Classification of Diseases, Ninth Revision, Clinical Modification; DISC-IV, Diagnostic Interview Schedule for Children-Version 4; I²-res (%), the percentage of the residual variation that is attributable to between-study heterogeneity.

Table 4 shows the results of multivariate regression analyses. The following factors remained significant: years of data collection, region, and age of participants. Specifically, consistent with univariate regression results, ADHD prevalence was lowest in the first 10 years (1980–1990) and significantly increased in a spanning period of next 3 decades, and school-aged children and adolescents (*β* = 0.19, *P* < 0.001) and preschoolers combined with school-aged children and adolescents (*β* = 0.16, *P* = 0.003) yielded significantly higher prevalence estimates than preschoolers. In addition, the rural areas showed significantly higher prevalence estimates than urban areas (*β* = 0.34, *P* = 0.009).

**Table 4:** The associations between study characteristics and the ADHD prevalence rate.

Study characteristics	*β*	95% CI
**Years of data collection (1980–1990 as index)**		
1991–2000	0.21*	0.09–0.32
2001–2010	0.20*	0.12–0.28
2011–2016	0.19*	0.11–0.28
**Geographical location (Mainland China as index)**		
Hong Kong	−0.05	−0.23–0.13
Taiwan	−0.07	−0.18–0.04
Region (urban areas as index)		
Rural areas	0.34*	0.09–0.59
Urban, & rural areas	−0.02	−0.09–0.06
**Age of participants (preschoolers as index)**		
School-aged children and adolescents	0.19*	0.10–0.27
Preschoolers, & school-aged children and adolescents	0.16*	0.06–0.26
**Sample size (≤2,000 as index)**		
2,000–5,000	0.03	−0.05–0.10
>5,000	−0.07	−0.14–0.01
**Procedure of screening and/or diagnosis (screening as index)**		
Diagnosis	0.09	−0.02–0.20
Screening, & diagnosis	0.06	−0.004–0.12

I²-res (%), the percentage of the residual variation that is attributable.

to between-study heterogeneity. The overall I²-res (%) is 98.22.

^*^P < 0.05.

## Discussion

We identified 67 original studies conducted in Mainland China, Hong Kong, and Taiwan from 1980 to 2016, covering 642,266 children and adolescents. Our prevalence estimate (6.3%) was lower than the 7.2% reported in a worldwide systematic review that included 175 studies from 1977 to 2013^25^. This discrepancy can be associated with the fact that a handful of Chinese studies (15 studies) were selected by Thomas *et al*.^25^. Meanwhile, our prevalence estimate was pronouncedly higher than the 3.4% reported in another systematic review study that included 48 studies from 1985 to 2012, with only one Chinese study^9^. Those worldwide ADHD systematic reviews were mainly based on original investigations conducted in Western countries and published in English. Therefore, they neglected a substantial proportion of Chinese investigations and publications, further bringing about both selection bias and publication bias. On the contrary, our pooled ADHD prevalence was highly representative of Chinese children and adolescents, an apparent advantage to generate better population-based benchmarks for Chinese professionals and the public, and to be beneficial for the accurate estimation of the worldwide ADHD prevalence.

Our study revealed that ADHD prevalence in Chinese children and adolescents arose over time, with slight fluctuations. Even though recent worldwide systematic reviews with meta-analyses showed no evidence of the ascent in the number of children who met the standard diagnostic criteria over the past three decades^25,26^, a roster of previous studies that employed the data collected in the USA, UK, and Canada from the 1990s to 2000s exhibited a time trend of mounting ADHD diagnoses and prescriptions of medications for ADHD treatment^27–32^. Similarly, the present study showed the investigations implemented from the next 3 decades reported a higher ADHD prevalence rate than those from 1980–1990. The ascending academic pressure emanate from the fierce Chinese educational competition may be associated with the increase in the number of Chinese school-aged children and adolescents with ADHD symptoms.

We also found that the rates reported by both parents and teachers were higher than those reported by either parents or teachers, corresponding to the stereotype that Chinese children should obey their both parents and teachers, and very active children are generally considered to be either badly behaved or hyperactive, especially in the context of the rising recognition of ADHD in recent years. Additionally, the result from the present study that school-aged children and adolescents had higher prevalence estimates than preschoolers may be explained by the phenomenon that elementary school teachers in China start to demand students follow more behavioral norms, e.g., sitting still in a classroom arrayed with desks and chairs, or standing in line. However, since the mixed-age participants in different grades mostly constituted the selected samples in our review, we were limited to divide the school-aged children and adolescents into elementary school, middle school, and high school children groups to discern the differences in ADHD prevalence among those subgroups. Consistent with the result that children from low-socioeconomic status (SES) backgrounds were more likely to exhibit ADHD symptoms than their peers from high-SES backgrounds^33,34^, children in rural areas showed a significantly higher ADHD prevalence than their counterparts in urban areas in our study. Nevertheless, caution should be taken in drawing conclusions in that only one selected study consisted of the sample solely from rural areas.

While our systematic review included studies specifically conducted in Mainland China, Hong Kong, and Taiwan, no difference was detected in the ADHD prevalence estimates among the three regions after controlling for other factors of the heterogeneity across studies. This finding corroborated the limited function of geographic location in the large variability of ADHD prevalence estimates which was found in the previous review with worldwide samples^11^. Nonetheless, it may not be neglected that remarkable differences in the socioeconomic development among the three regions during the last three decades may greatly impact the ADHD prevalence estimates. The previous worldwide systematic review also suggested that the heterogeneity of methodological characteristics may have caused the differences in ADHD prevalence in different locations^11^. Our review indicated the similar findings that variations of the sample size, study design and screening/diagnostic criteria among the three regions explained the regional differences in prevalence estimates. For instance, although most included studies were conducted in Mainland China, studies in Taiwan had the largest number of participants, and they were more weighted in our meta-analyses. Whereas most studies in Mainland China and Hong Kong were cross-sectional, most studies in Taiwan were longitudinal. In general, compared to studies from Mainland, which wide range of screening/diagnostic criteria were used, most investigators from Hong Kong and Taiwan selected DSM and ICD-based criteria to define the ADHD.

### Limitations

First, the literature published in the local languages of Hong Kong and Taiwan was not included in our review. Second, the high heterogeneity across studies and publication bias may weaken our ability to precisely estimate the ADHD prevalence among Chinese children and adolescents. Specifically, the pronounced variations in the procedures of screening and/or diagnosis and associated criteria across the studies raised the incomparability across the original ADHD prevalence rates, and thus caused the uncertainty to our pooled prevalence estimates. Third, the ADHD prevalence estimates found in our subgroup meta-analyses cannot adequately discern the differences in economic situations among different Urban and rural areas, and the subgroup estimates cannot be generalized to the only rural areas.

## Conclusions

This is one of the few comprehensive systematic reviews of ADHD prevalence estimates among Chinese children and adolescents in Mainland China, Hong Kong, and Taiwan over the past three decades. The prevalence estimates of ADHD among children in Mainland China and Hong Kong are similar and consistent with the reported rate in previous reviews. However, Taiwan has significantly lower prevalence than other regions. Even though our results should be interpreted with caution because of the large variability found in the analyses. Moreover, our findings suggest that the geographic location plays a limited role in the heterogeneity of ADHD prevalence estimates in Chinese children. Instead, the variability may be primarily explained by the methodological characteristics of studies, years of data collection, and participants’ socioeconomic backgrounds. Our analyses also indicate that high-quality studies, such as cohort studies or repeated cross-sectional studies, are required to assess the true trend of ADHD prevalence.

## Methods and Materials

### Literature Search

A search of the literature published in English was performed using PubMed, Embase, PsycINFO, and Web of Science databases. The literature published in Chinese was searched using the China National Knowledge Infrastructure, VIP, WANFANG DATA, and China Science Periodical Database databases. These four Chinese databases include most of the articles published in Chinese among Mainland China, Hong Kong, and Taiwan. The year of publication was confined between 1978 and 2016, because the World Health Organization enacted the International Classification of Diseases, Ninth Edition (ICD-9) in which hyperkinetic disorder was defined (an alternative name for ADHD) in 1978. The search strategy was composed of search fragments of population (i.e. children and adolescent), disease condition (i.e. attention deficit disorder with hyperactivity), outcome (i.e. prevalence) and geographic location (i.e. China). Four investigators (L.A.N., X.Y.W., Y.Q., and T.L.) used the same search strategies for all databases when conducting the literature search (Appendix 1).

### Study Inclusion and Exclusion Criteria

Three authors (L.A.N., X.Y.W., and T.L.) worked on the selection, inclusion, and exclusion criteria. Each author independently conducted a literature search, reviewed abstracts for further full-text reviews, and selected eligible studies according to the preset criteria. Studies with incomplete data or disagreements could not be included in the final analyses unless the three authors reached a consensus.

The selection criteria were: (1) original prevalence studies were conducted in the Mainland of China, Hong Kong, or Taiwan; (2) participants aged 18 years old or younger; (3) participants were screened for and/or diagnosed with ADHD; (4) any of the following assessment tools for ADHD was applied: Conners’ Parent Rating Scale (Conners), Conners’ Teacher Rating Scale (Conners), DSM-III, Diagnostic and Statistical Manual of Mental Disorders, Revised Third Edition (DSM-III-R), DSM-IV, International Classification of Diseases, Ninth Revision, Clinical Modification (ICD-9-CM), International Classification of Diseases, Tenth Edition (ICD-10), Chinese Classification and Diagnosis of Mental Diseases, Second Edition (CCMD-II), Chinese Classification and Diagnosis of Mental Diseases, Revised Second Edition (CCMD-II-R), Chinese Classification and Diagnosis of Mental Diseases, Third Edition (CCMD-III), Diagnostic Interview Schedule for Children-Version 4 (DISC-IV), and others (e.g., standard questionnaires/interviews/clinical checks).

Inclusion criteria were: (1) the epidemiological survey must have been conducted in the Mainland of China, Hong Kong, or Taiwan; (2) the study must specify the ADHD prevalence rate, rather than that of individual ADHD symptoms, e.g., attention deficit or hyperactivity; (3) participants must have been children or adolescents younger than 18 years old who were native Chinese/Hong Kongese/Taiwanese; (4) the study must have used any of the following standardized assessment tools for ADHD screening and/or diagnosis: Conners, DSM-III, DSM-III-R, DSM-IV, ICD-9-CM, ICD-10, CCMD-II, CCMD-II-R, CCMD-III, DISC-IV, others (e.g., standard questionnaires/interviews/clinical checks) or possible combinations; (5) the study must be population based; (6) the sample size was at least 500; (7) the article must be written in Chinese or English.

Exclusion criteria were: (1) participants were over 18 years old; (2) participants were migrant children or adolescents; (3) none of the following standardized tools was employed: Conners, DSM-III/III-R/IV, ICD-9-CM/-10, CCMD-II/-II-R/III, DISC-IV or others (e.g., standard questionnaires/interviews/clinical checks); 4) the study was clinic based or patient based; 5) the sample size was less than 500, considering potential lower power due to small sample size.

### Data extraction

The following key variables were extracted: 1) title of article; 2) years of data collection (the publication year was used as a proxy for studies without this information); 3) geographical locations (Mainland China, Hong Kong, and Taiwan); 4) time frame (referring to the period of data collection; 5) regions (rural area, urban area, or combination of rural and urban areas); 6) age of participants; 7) sample size; 8) procedure of screening and/or diagnosis; 9) screening criteria; 10) source of screening information; 11) diagnostic criteria; 12) overall ADHD prevalence rate; 13) gender-specific ADHD prevalence rates; 14) number of participants with ADHD; 15) gender-specific numbers of participants with ADHD. All the variables were collected and double checked by 2 reviewers (L.A.N., and X.Y.W.), with a third reviewer (T.L.) acting as arbitrator. The description of included studies is shown in the Table 1.

### Risk of Bias Assessment

Two reviewers (L.A.N. and T. L.) assessed the risk of bias for each included study using a reliable Risk of Bias Tool for prevalence studies developed by Hoy *et al*.^35^. Each included study was judged by 10 items that assess measurement bias, selection bias, and bias related to the analysis (all rated as either high or low risk) and an overall assessment of risk of bias rated as low, moderate, or high risk. The more criteria were met, the lower the risk of bias. If the text was unclear, a high risk of bias was then recorded. A study was considered to have a high overall risk of bias if 3 criteria or less were met, moderate risk of bias if 4 to 6 criteria were met, and low risk of bias if 7 to 10 criteria were met.

### Data Analysis

**Figure 3:**
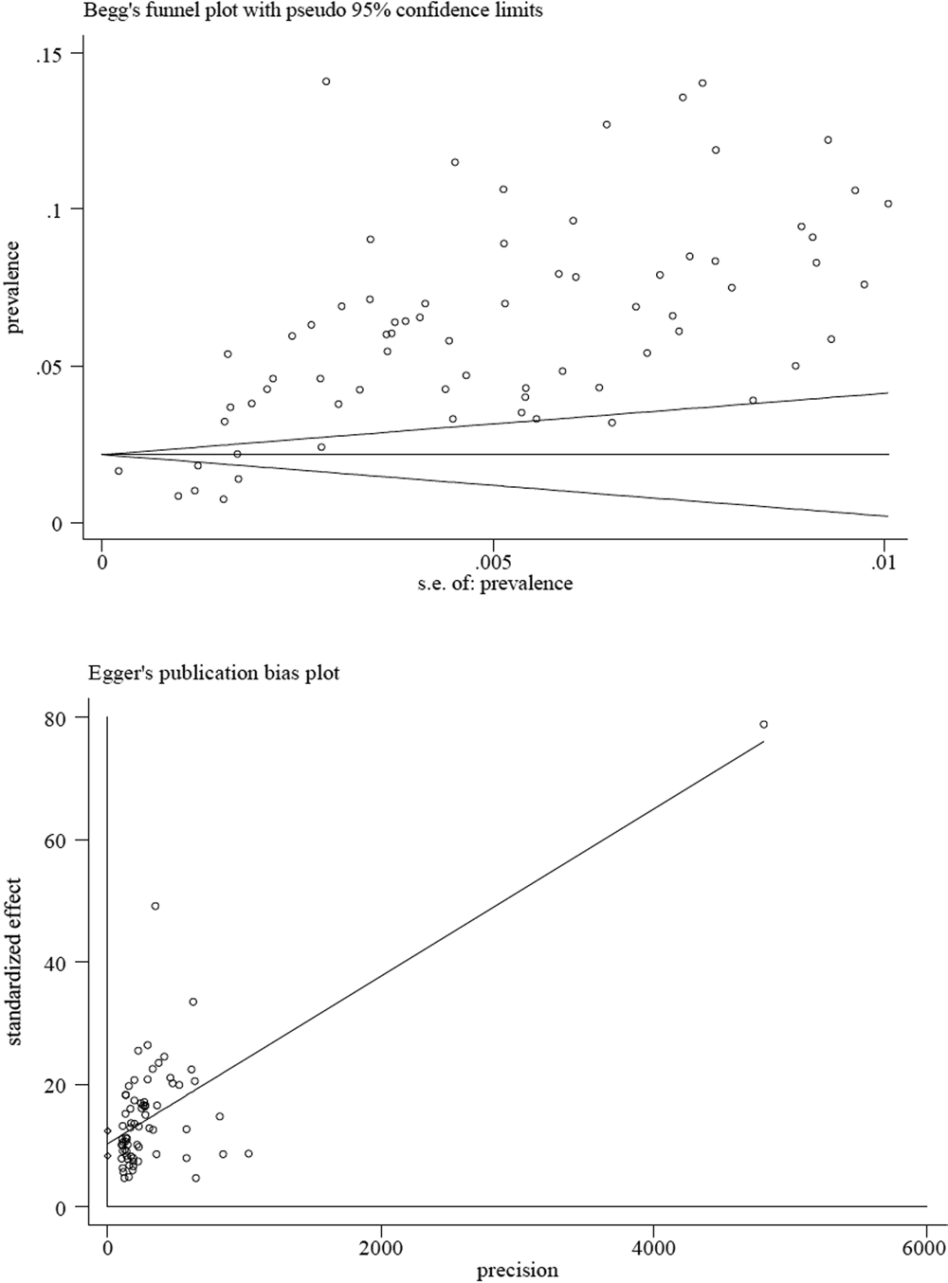
Begg’s and Egger’s funnel plots for analysis of publication bias.

To minimize the effects of extreme prevalence rates on the overall estimates, we stabilized the variance of the study-specific prevalence with the Freeman-Tukey double arcsine transformation^36^ in both univariate and multivariate models. We applied Begg’s Test and Egger’s test^37^ to test publication bias. Inferred from the funnel and bias plots (Fig. 3), we performed the trim and fill method. The results indicated that no additional prevalence study was needed to adjust for the publication bias^38^. Funnel plot asymmetry does not necessarily indicate publication bias (PB) in proportion studies^39^. The quantity *I*^2^ was used to detect the heterogeneity of this meta-analysis^40^. Next, we fitted a random-effect model to estimate the overall and subgroup pooled prevalence of ADHD using untransformed prevalence rates. To further explore the potential sources of heterogeneity, we conducted the random-effect meta-regression analyses using transformed prevalence rates. Dummy variables were used in our univariate and multivariate meta-regression analyses. All data analyses were performed using Stata 14.0 (Stata Corp, College Station, TX). A two-tailed *P* value of less than 0.05 was considered statistically significant.

Both univariate and multivariate meta-regression analysis were carried out. In each model, for categorical variable, one group was set as the reference group according to the purpose of analysis. In the final multivariate meta-regression model, the variable time frame was excluded due to the insufficient supportive literature regarding the role of time frame for data collection in the heterogeneity of ADHD prevalence findings. Additionally, the variable procedure of screening and/or diagnosis was included in the final model instead of screening criteria or diagnostic criteria because placing the latter variables in the multivariate regression model would greatly reduce the number of samples and decrease the precision. Additionally, 6 studies did not report their sources of screening information, thus the variable source of information was dropped as well. In summary, the following covariates were finally examined in the multivariate model using the restricted maximum likelihood estimator: years of data collection, geographic location, region, age of participants, sample size, and procedure of screening and/or diagnosis. Stepwise was used to select the significant variables to the model.

### Data availability

The datasets generated and analyzed during the current study are available from the corresponding author on reasonable request.

## Electronic supplementary material

Appendix 1.Search Strategy

Appendix 2. PRISMA checklist
