# Supplementary material for: The Prevalence of Attention Deficit/Hyperactivity Disorder among Chinese Children and Adolescents
Source: Sci Rep. 2018 Aug 16;8:11169. doi: 10.1038/s41598-018-29488-2 (PMC6095841; doi:10.1038/s41598-018-29488-2)
Supplement: Supplementary file 1 — Appendix 1.Search Strategy [file 41598_2018_29488_MOESM1_ESM.pdf]

The Prevalence of Attention Deficit/Hyperactivity Disorder among Chinese Children and Adolescents

Anni Liu[1], Yunwen Xu[2], Qiong Yan[1], Lian Tong[1*]

1 School of Public Health, Fudan University/Key Laboratory Public Health Safety, Chinese Ministry of Education, Shanghai, China

2 Department of Epidemiology, Johns Hopkins University Bloomberg School of Public Health, Baltimore, Maryland, United States

*** Corresponding Author:**

Lian Tong

Department of Maternal, Child and Adolescent health, School of Public Health, Fudan University / Key Laboratory Public Health Safety, Chinese Ministry of Education

P.O. Box 244, 138 Yixueyuan Road, Shanghai 200032, China

Tel: +86 21 6564 2996;

Fax: +86 21 6564 2996

Email: ltong@fudan.edu.cn

Appendix 1. Search Strategy

The search strategy was in English for the PubMed, Embase, PsycINFO and Web of Science databases and the corresponding search strategy was in Chinese for the China National Knowledge Infrastructure, VIP, WANFANG DATA, and China Science Periodical databases. The search strategies included at least one search item from four search fragments shown as below:

1) "attention deficit disorders with hyperactivity" OR "attention deficit hyperactivity disorders" OR "hyperkinetic syndrome" OR "minimal brain dysfunction" OR ADD OR ADHD OR "hyperkinetic disorder";
2) child* OR adolescen* OR teen* OR youth* OR preschooler*;
3) prevalence* OR survey* OR epidemi* OR investigat* OR surveillance;
4) China OR Chinese OR "People's Republic of China" OR PRC OR "Mainland China" OR Taiwan OR "Republic of China" OR ROC OR Formosa OR Taiwanese OR "Taiwanese people" OR "Hong Kong" OR "Hong Kong Special Administrative Region of the People's Republic of China" OR "Hong Kong people" OR Hong Kongers OR "Hong Kongese" OR "Hong Kongers" OR Hong Kongese.

Appendix 2. PRISMA Flowchart

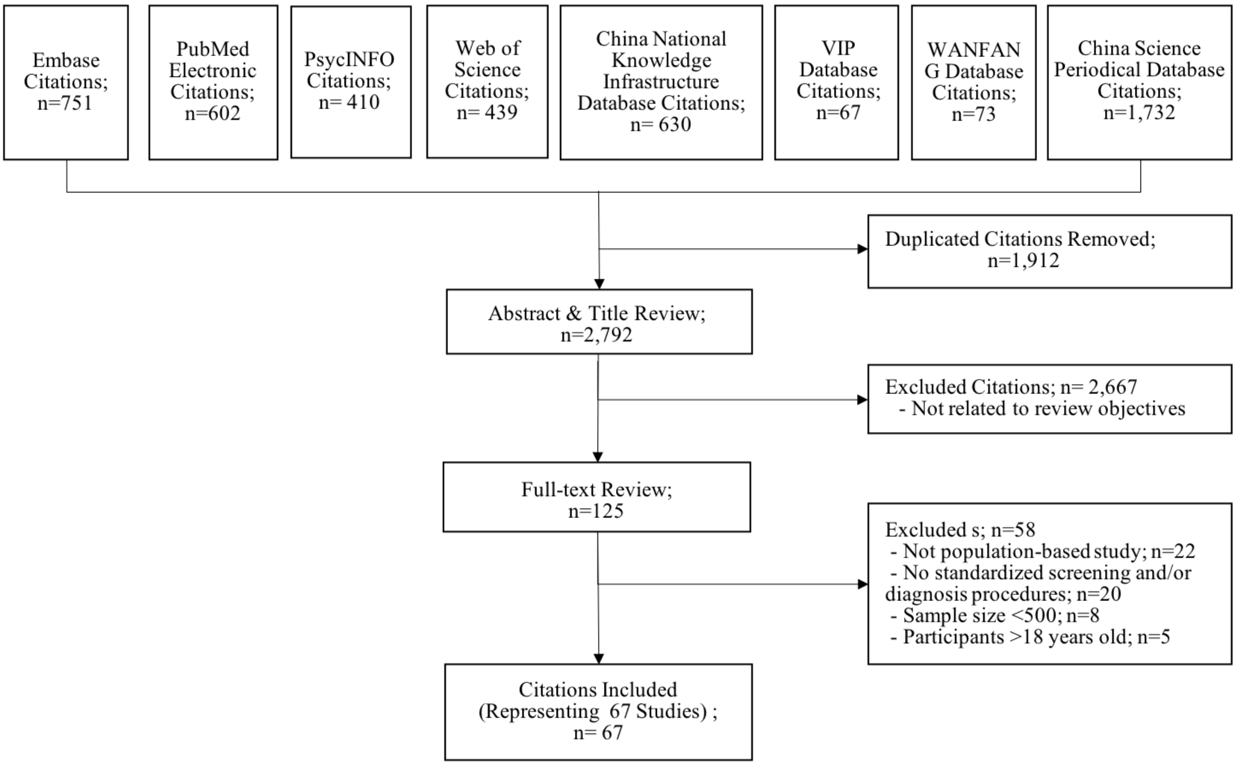

Figure 1. PRISMA Flowchart
