# Supplementary material for: The Prevalence of Attention Deficit/Hyperactivity Disorder among Chinese Children and Adolescents
Source: Sci Rep. 2018 Aug 16;8:11169. doi: 10.1038/s41598-018-29488-2 (PMC6095841; doi:10.1038/s41598-018-29488-2)
Supplement: Supplementary file 2 — Appendix 2. PRISMA checklist [file 41598_2018_29488_MOESM2_ESM.pdf]

The Prevalence of Attention Deficit/Hyperactivity Disorder among Chinese Children and Adolescents

Anni Liu[1], Yunwen Xu[2], Qiong Yan[1], Lian Tong[1*]

1 School of Public Health, Fudan University/Key Laboratory Public Health Safety, Chinese Ministry of Education, Shanghai, China

2 Department of Epidemiology, Johns Hopkins University Bloomberg School of Public Health, Baltimore, Maryland, United States

*** Corresponding Author:**

Lian Tong

Department of Maternal, Child and Adolescent health, School of Public Health, Fudan University / Key Laboratory Public Health Safety, Chinese Ministry of Education

P.O. Box 244, 138 Yixueyuan Road, Shanghai 200032, China

Tel: +86 21 6564 2996;

Fax: +86 21 6564 2996

Email: ltong@fudan.edu.cn

PRISMA 2009 Checklist

Section/topic	#	Checklist item	Reported on page #
**TITLE**			
Title	1	The Prevalence of Attention Deficit/Hyperactivity Disorder among Chinese Children and Adolescents: A systematic review and meta-analysis	1
**ABSTRACT**			
Structured summary	2	*Context*: Updating the worldwide prevalence estimates of attention-deficit hyperactivity disorder (ADHD) has significant applications for the further study of ADHD. However, previous reviews included few samples of Chinese children and adolescents. *Objectives*: To conduct a systematic review of ADHD prevalence in Mainland China, Hong Kong, and Taiwan to determine the possible causes of the varied estimates in Chinese samples and to offer a reference for computing the worldwide pooled prevalence. *Data Sources*: We searched for PubMed, Embase, PsycINFO, Web of Science, China National Knowledge Infrastructure, VIP, WANFANG DATA, and China Science Periodical Database databases with time and language restrictions. *Results*: A total of 67 studies covering 642,266 Chinese children and adolescents were included. The prevalence estimates of ADHD in Mainland China, Hong Kong, and Taiwan were 6.5%, 6.4%, and 4.2%, respectively, with a pooled estimate of 6.3%. Multivariate meta-regression analyses indicated that the year of data collection, age, and family socioeconomic status of the participants were significantly associated with the prevalence estimates. *Conclusions*: Our findings suggest that geographic location plays a limited role in the large variability of ADHD prevalence estimates. Instead, the variability may be explained primarily by the years of data collection, and children's socioeconomic backgrounds, and methodological characteristics of studies.	2
**INTRODUCTION**			
Rationale	3	Concerns have been raised regarding the true prevalence of ADHD among children, the knowledge of which is critical for further service planning, resource allocation, training, and research priorities[8]. In the last few decades, a host of investigators have made substantial efforts to determine the prevalence of ADHD. However, previous systematic reviews seldom selected a sufficient proportion of studies conducted among Asian children and adolescents, and were especially lacking of Chinese samples, despite the fact that China has the largest number of children and adolescents in the world. It is clear that an estimated ADHD prevalence from one location fails to represent the overall prevalence among Chinese children, while a systematic understanding of the ADHD prevalence estimates in Chinese children and adolescents may provide a better insight into the overall and subgroup distribution and etiology of ADHD under different social and cultural backgrounds. Furthermore, a meta-analysis that computes the prevalence estimates of ADHD in the three regions will offer the supportive data for the accurate prediction of the worldwide pooled prevalence.	3-5
Objectives	4	1) to estimate the overall and subgroup prevalence estimates of ADHD among children and adolescents in Mainland China, Hong Kong, and Taiwan from 1980 to 2016; 2) to analyze the trends of ADHD prevalence in the three locations in a period spanning the past 3 decades to aid in predicting future trends; and 3) to explore the possible causes of the varied prevalence estimates.	5

METHODS			
Protocol and registration	5	Not applicable	
Eligibility criteria	6	The selection criteria were: 1) original prevalence studies were conducted in the Mainland of China, Hong Kong, or Taiwan; 2) participants aged 18 years old or younger; 3) participants were screened for and/or diagnosed with ADHD; 4) any of the following assessment tools for ADHD was applied: Conners' Parent Rating Scale (Conners), Conners' Teacher Rating Scale (Conners), DSM-III, Diagnostic and Statistical Manual of Mental Disorders, Revised Third Edition (DSM-III-R), DSM-IV, International Classification of Diseases, Ninth Revision, Clinical Modification (ICD-9-CM), International Classification of Diseases, Tenth Edition (ICD-10), Chinese Classification and Diagnosis of Mental Diseases, Second Edition (CCMD-II), Chinese Classification and Diagnosis of Mental Diseases, Revised Second Edition (CCMD-II-R), Chinese Classification and Diagnosis of Mental Diseases, Third Edition (CCMD-III), Diagnostic Interview Schedule for Children-Version 4 (DISC-IV), and others (e.g., standard questionnaires/interviews/clinical checks).<br>Inclusion criteria were: 1) the epidemiological survey must have been conducted in the Mainland of China, Hong Kong, or Taiwan; 2) the study must specify the ADHD prevalence rate, rather than that of individual ADHD symptoms, e.g., attention deficit or hyperactivity; 3) participants must have been children or adolescents younger than 18 years old who were native Chinese/Hong Kongese/Taiwanese; 4) the study must have used any of the following standardized assessment tools for ADHD screening and/or diagnosis: Conners, DSM-III, DSM-III-R, DSM-IV, ICD-9-CM, ICD-10, CCMD-II, CCMD-II-R, CCMD-III, DISC-IV, others (e.g., standard questionnaires/interviews/clinical checks) or possible combinations; 5) the study must be population based; 6) the sample size was at least 500; 7) the article must be written in Chinese or English.<br>Exclusion criteria were: 1) participants were over 18 years old; 2) participants were migrant children or adolescents; 3) none of the following standardized tools was employed: Conners, DSM-III/III-R/IV, ICD-9-CM/-10, CCMD-II/-II-R/III, DISC-IV or others (e.g., standard questionnaires/interviews/clinical checks); 4) the study was clinic based or patient based; 5) the sample size was less than 500, considering potential lower power due to small sample size.	14-16
Information sources	7	A search of the literature published in English was performed using PubMed, Embase, PsycINFO, and Web of Science databases. The literature published in Chinese was searched using the China National Knowledge Infrastructure, VIP, WANFANG DATA, and China Science Periodical Database databases. The year of publication was confined between 1978 and 2016.	14
Search	8	The English literature was searched through PubMed, Embase, PsycINFO and Web of Science databases and the literature in Chinese for searched by China National Knowledge Infrastructure, VIP, WANFANG DATA, and China Science Periodical databases. The search strategies included at least one search item from four search fragments shown as below:<br>1) "attention deficit disorders with hyperactivity" OR "attention deficit hyperactivity disorders" OR "hyperkinetic syndrome" OR "minimal brain dysfunction" OR ADD OR ADHD OR "hyperkinetic disorder";<br>2) child* OR adolescen* OR teen* OR youth* OR preschooler*;<br>3) prevalence* OR survey* OR epidemi* OR investigat* OR surveillance;	Appendix 1

		4) China OR Chinese OR "People's Republic of China" OR PRC OR "Mainland China" OR Taiwan OR "Republic of China" OR ROC OR Formosa OR Taiwanese OR "Taiwanese people" OR "Hong Kong" OR "Hong Kong Special Administrative Region of the People's Republic of China" OR "Hong Kong people" OR Hong Kongers OR "Hong Kongese" OR "Hong Kongers" OR Hong Kongese.	
Study selection	9	Inclusion criteria were: 1) the epidemiological survey must have been conducted in the Mainland of China, Hong Kong, or Taiwan; 2) the study must specify the ADHD prevalence rate, rather than that of individual ADHD symptoms, e.g., attention deficit or hyperactivity; 3) participants must have been children or adolescents younger than 18 years old who were native Chinese/Hong Kongese/Taiwanese; 4) the study must have used any of the following standardized assessment tools for ADHD screening and/or diagnosis: Conners, DSM-III, DSM-III-R, DSM-IV, ICD-9-CM, ICD-10, CCMD-II, CCMD-II-R, CCMD-III, DISC-IV, others (e.g., standard questionnaires/interviews/clinical checks) or possible combinations; 5) the study must be population based; 6) the sample size was at least 500; 7) the article must be written in Chinese or English.	14
Data collection process	10	Three authors worked together on the selection, inclusion, and exclusion criteria. Each author independently conducted a literature search, reviewed abstracts for further full-text reviews, and selected eligible studies according to the preset criteria. Studies with incomplete data or disagreements could not be included in the final analyses unless the three authors reached a consensus. All the variables were collected and double checked by 2 authors.	16
Data items	11	The following key variables were extracted:<br>1) title of article; 2) years of data collection (the publication year was used as a proxy for studies without this information); 3) geographical locations (Mainland China, Hong Kong, and Taiwan); 4) time frame (referring to the period of data collection; 5) regions (rural area, urban area, or combination of rural and urban areas); 6) age of participants; 7) sample size; 8) procedure of screening and/or diagnosis; 9) screening criteria; 10) source of screening information; 11) diagnostic criteria; 12) overall ADHD prevalence rate; 13) gender-specific ADHD prevalence rates; 14) number of participants with ADHD; 15) gender-specific numbers of participants with ADHD.	16
Risk of bias in individual studies	12	Two reviewers (L.A.N. and T. L.) assessed the risk of bias for each included study using a reliable Risk of Bias Tool for prevalence studies developed by Hoy et al (2012)[35]. Each included study was judged by 10 items that assess measurement bias, selection bias, and bias related to the analysis (all rated as either high or low risk) and an overall assessment of risk of bias rated as low, moderate, or high risk. The more criteria were met, the lower the risk of bias. If the text was unclear, a high risk of bias was then recorded. A study was considered to have a high overall risk of bias if 3 criteria or less were met, moderate risk of bias if 4 to 6 criteria were met, and low risk of bias if 7 to 10 criteria were met.	17
Summary measures	13	A multivariate meta-regression model was used to examine the relevant factors (beta coefficient). Overall pooled prevalence and sub-group prevalence rates were calculated.	17
Synthesis of results	14	Describe the methods of handling data and combining results of studies, if done, including measures of consistency (e.g., I^2) for each meta-analysis.<br>The quantity I2 was used to detect the heterogeneity of this meta-analysis.	17

PRISMA 2009 Checklist

Section/topic	#	Checklist item	Reported on page #
Risk of bias across studies	15	We applied Begg's Test and Egger's test[37] to test publication bias. Inferred from the funnel and bias plots (Figure 1), we performed the trim and fill method.	17
Additional analyses	16	Both univariate and multivariate meta-regression analysis were carried out. Stepwise was used to select the significant variables to the model.	17-18
**RESULTS**			
Study selection	17	We screened 4704 abstracts, reviewed 125 full-text articles, and selected 67 studies for the final systematic review. Of these, 13 were published in English and 54 were published in Chinese. Figure 2 presents the flowchart of study selection.	5; Figure 2
Study characteristics	18	Table 1 displays the characteristics of the articles included in this systematic review.	5-6; 29-32
Risk of bias within studies	19	Although all estimates from 67 studies were at moderate or low risk of bias, only 1 estimate met all 10 criteria, and 65% were at low risk of bias. The majority of estimates rated poorly for the representativeness of the national population (93%), and the strict measurement of the reliability and validity of the study instrument (85%). Besides, most estimates did not collect ADHD diagnostic information directly from children or adolescents (93%). Summary statistics for risk of bias for estimates are provided in Table 1.	6-7; 29-32
Results of individual studies	20	A forest plot was included in the text.	
Synthesis of results	21	The pooled prevalence of ADHD was 6.3% (95% confidence interval [CI], 5.7–6.9). Substantial heterogeneity across studies was detected (I2 = 99%; Q = 9121.98, df = 69, P < .001)	7-8
Risk of bias across studies	22	PInferred from the funnel and bias plots (Figure 1), we performed the trim and fill method. The results indicated that no additional prevalence study was needed to adjust for the publication bias38.	Figure 1
Additional analysis	23	The estimated rates in Mainland China, Hong Kong, and Taiwan were 6.5% (95% CI, 5.7–7.3), 6.4% (95% CI, 1.5–11.3), and 4.2% (95% CI, 3.2–5.2), respectively. ADHD was more common in boys (8.9%, 95% CI, 7.6–10.2) than in girls (4.0%, 95% CI, 3.4–4.7). The pooled prevalence rates were 5.5% (95% CI, 4.2–6.8) between 1980 and 1990, 6.9% (95% CI 4.2–9.6) between 1991 and 2000, 6.0% (95% CI, 5.2–6.7) between 2001 and 2010, and 6.7% (95% CI, 5.2–8.2) between 2011 and 2016. The pooled prevalence rate was 5.5% (95% CI, 3.3-7.7) for preschoolers, and 6.5% (95% CI, 5.5-7.4) for school-aged children and/or adolescents, while the overall prevalence of combining two age groups was 6.1% (95% CI, 5.1-7.2). The forest plot of the subgroup estimates is presented in Figure 3. Also see Table 2.<br>In univariate meta-regression analyses (Table 3), there was a significant increase in prevalence estimates in all three periods of 1991-2000 (β = 0.39, P<.001), 2001-2010 (β = 0.36, P<.001), and 2011-2016 (β = 0.38, P<.001) compared with the period of 1980-1990. The studies with combined samples from both Urban, & rural areas yielded significantly	8-9; Figure3; 33-37

		higher ADHD prevalence estimates than those with urban samples (β = 0.35, P < .001). Both school-aged children and/or adolescents (β = 0.37, P <.001) and preschoolers combined with school-aged children and/or adolescents (β = 0.37, P < .001) had significantly higher prevalence estimates than preschoolers. The larger sample sizes of 2000–5000 (β = 0.39, P < .001) or over 5,000 (β = 0.32, P < .001) generated significantly higher prevalence estimates than the sample sizes of less than 2000. There was a significant increase in prevalence estimates when the informants were parents (β = 0.37, P< .001) as well as both teachers and parents (β = 0.39, P < .001) compared with only teachers. The studies that underwent diagnostic procedure (β = 0.33, P < .001) or both screening and diagnostic procedures (β = 0.37, P < .001) displayed significantly higher prevalence estimates than those only with screening procedure. The studies employing Conners-based screening criteria yielded significantly lower estimates than those with other screening criteria, e.g., DSM-III/-III-R (β= 0.37, P = .019), DSM-IV/DISC-IV (β= 0.37, P < .001), Conners combined with DSM criteria (β = 0.44, P < .001). Compared to the studies conducted with the diagnostic criteria of DSM-III/-III-R, the studies using DSM-IV/DISC-IV (β= 0.37, P < .001), CCMD-II/II-R/III (β = 0.36, P = .001), DSM-IV combined with Conners (β = 0.42, P < .001) or ICD-9-CM (β = 0.25, P = .02) as the diagnostic criteria had significantly higher prevalence estimates.<br><br>Table 4 shows the results of multivariate regression analyses. The following factors remained significant: years of data collection, region, and age of participants. Specifically, consistent with univariate regression results, ADHD prevalence was lowest in the first 10 years (1980–1990) and significantly increased in a spanning period of next 3 decades, and school-aged children and/or adolescents (β = 0.19, P < .001) and preschoolers combined with school-aged children and/or adolescents (β = 0.16, P = .003) yielded significantly higher prevalence estimates than preschoolers. In addition, the rural areas showed significantly higher prevalence estimates than urban areas (β = 0.34, P = .009).	
**DISCUSSION**			
Summary of evidence	24	We identified 67 original studies conducted in Mainland China, Hong Kong, and Taiwan from 1980 to 2016, covering 642,266 children and adolescents. Our prevalence estimate (6.3%) …our pooled ADHD prevalence was highly representative of Chinese children and adolescents, an apparent advantage to generate better population-based benchmarks for Chinese professionals and the public, and to be beneficial for the accurate estimation of the worldwide ADHD prevalence.<br><br>Our study revealed that ADHD prevalence in Chinese children and adolescents arose over time, with slight fluctuations…The ascending academic pressure emanate from the fierce Chinese educational competition may be associated with the increase in the number of Chinese school-aged children and adolescents with ADHD symptoms.<br><br>We also found that the rates reported by both parents and teachers were higher than those reported by either parents or teachers, corresponding to the stereotype that Chinese children should obey their both parents and teachers, and very active children are generally considered to be either badly behaved or hyperactive, especially in the context of the rising recognition of ADHD in recent years. Additionally, the result from the present study that school-aged children and/or adolescents had higher prevalence estimates than preschoolers may be explained by the phenomenon that elementary school teachers in China start to demand students follow more behavioral norms, e.g., sitting still in a classroom arrayed with desks and chairs, or standing in line.<br><br>While our systematic review included studies specifically conducted in Mainland China, Hong Kong, and Taiwan, no	9-12

		difference was detected in the ADHD prevalence estimates among the three regions after controlling for other factors of the heterogeneity across studies. This finding corroborated the limited function of geographic location in the large variability of ADHD prevalence estimates which was found in the previous review with worldwide samples[11]. The previous worldwide systematic review also suggested that the heterogeneity of methodological characteristics may have caused the differences in ADHD prevalence in different locations[11]. Our review indicated the similar findings that variations of the sample size, study design and screening/diagnostic criteria among the three regions explained the regional differences in prevalence estimates…	
Limitations	25	First, the literature published in the local languages of Hong Kong and Taiwan was not included in our review. Second, the high heterogeneity across studies and publication bias may weaken our ability to precisely estimate the ADHD prevalence among Chinese children and adolescents. Specifically, the pronounced variations in the procedures of screening and/or diagnosis and associated criteria across the studies raised the incomparability across the original ADHD prevalence rates, and thus caused the uncertainty to our pooled prevalence estimates. Third, the ADHD prevalence estimates found in our subgroup meta-analyses cannot adequately discern the differences in economic situations among different Urban and rural areas, and the subgroup estimates cannot be generalized to the only rural areas.	12-13
Conclusions	26	This is one of the few comprehensive systematic reviews of ADHD prevalence estimates among Chinese children and adolescents in Mainland China, Hong Kong, and Taiwan over the past three decades. The prevalence estimates of ADHD among children in Mainland China and Hong Kong are similar and consistent with the reported rate in previous reviews. However, Taiwan has significantly lower prevalence than other regions, though our results should be interpreted with caution because of the large variability found in the analyses. Moreover, our findings suggest that the geographic location plays a limited role in the heterogeneity of ADHD prevalence estimates in Chinese children. Instead, the variability may be primarily explained by the methodological characteristics of studies, years of data collection, and participants' socioeconomic backgrounds. Our analyses also indicate that high-quality studies, such as cohort studies or repeated cross-sectional studies, are required to assess the true trend of ADHD prevalence.	13
**FUNDING**			
Funding	27	All phases of this study were funded by the National Natural Science Foundation of China (Grant No. 81402693), Shanghai Pujiang Program (Grant No.14PJC012) and Award from Shanghai Municipal Health Bureau (Grant No. 15GWZK0402). The authors thank Dr. Jelena Kolic from The University of British Columbia for her contribution to the manuscript revision. Competing financial interests: The authors declare no competing financial interest.	28

From: Moher D, Liberati A, Tetzlaff J, Altman DG, The PRISMA Group (2009). Preferred Reporting Items for Systematic Reviews and Meta-Analyses: The PRISMA Statement. PLoS Med 6(7): e1000097. doi:10.1371/journal.pmed1000097

For more information, visit: **www.prisma-statement.org**.
